# A simple adaptive difference algorithm with CO_2_ measurements for evaluating plant growth under environmental fluctuations

**DOI:** 10.1186/s13104-022-05929-0

**Published:** 2022-02-14

**Authors:** Hiroki Gonome, Jun Yamada, Norito Nishimura, Yuta Arai, Minoru Hirai, Naoki Kumagai, Uma Maheswari Rajagopalan, Takahiro Kono

**Affiliations:** 1 Department of Mechanical System Engineering, Yamagata University, Yamagata, 992-8510 Japan; 2 Department of Mechanical Engineering, Shibaura Institute of Technology, 3-7-5 Toyosu, Koto-ku, Tokyo, 135-8548 Japan

**Keywords:** Plant growth, CO_2_ gas-exchange system, Adaptive method, Environmental fluctuations, Photosynthesis, Pulsed light, Arugula

## Abstract

**Objective:**

The aim of this study is to demonstrate an adaptive method that is robust toward environmental fluctuations and provides a real-time measure of plant growth by measuring CO_2_ consumption. To verify the validity of the proposed method, the relation between the plant growth and variation in light conditions with a closed experimental system was investigated.

**Results:**

The proposed method was used to measure the photosynthetic rate induced by photosynthetic photon flux density (PPFD) and to evaluate plant growth under continuous and pulsed light in arugula plants. The PPFD-dependent change in photosynthetic rate was measured. And in the condition range of 200–10,000 μs pulse period and 50% duty ratio of pulsed light, there was no change in the growth rate of plants assuming the same PPFD as continuous light. These experiments showed the validity of the adaptive method in removing environmental fluctuations without precise control of temperature and humidity.

**Supplementary Information:**

The online version contains supplementary material available at 10.1186/s13104-022-05929-0.

## Introduction

In Japan, plant factories are becoming increasingly common for commercial vegetable production, mainly because of their efficiency and flexibility in terms of commercial horticulture. Plant factories ensure highly efficient plant production by controlling parameters, such as room light intensity and wavelength spectrum to be optimal for plant production [1–9]. However, the enormity of the environmental parameters that need to be controlled requires the experimental evaluation over a long period of time for each kind of the plant [10]. Therefore, there is an immediate need for an environmentally robust experimental evaluation method to determine the optimal conditions for a particular plant species.

Many recent studies have considered different evaluation methods for environmental variations. Dong et al. [11] investigated the influence of environmental conditions on the efficiency of wheat production. However, their analyses were conducted after the wheat had finished growing. Chen et al. [12] and Kim et al. [13], who proposed image processing methods for evaluating plant growth, were similarly constrained because the evaluation was once again conducted after plant growth had been completed. In these methods, the evaluation takes at least several months to wait for the plant growth to finish and reach maturity.

To measure the plant growth in real-time, gas-exchange systems, which measure photosynthesis by uptake of CO_2_ or consumption of O_2_, have come a long way [14–19]. There are three main types of gas-exchange systems: open [15], semi-closed [16–18] and closed systems [19]. The open system continuously renews the air inside the chamber while measuring the gas concentration at the entry and exit airstreams. In most open chamber systems with air supply, there is a constant overpressure within the chamber to keep the structure inflated. Furthermore, the speed of steady air flow is often higher than that of the natural wind and a suitable ventilation rate is difficult to attain. Also, for the closed systems, humidity and temperature have to be controlled because these environmental factors affect the photosynthesis of the plant. Thus, for the gas-exchange systems, it is necessary to control the environmental factors precisely during the experiment to evaluate the plant growth. In order to find the optimal environmental conditions for many types of plants, it is necessary to create a simple, robust, and a speedy method that does not require complicated and precise equipments.

In our research, we propose an adaptive method to measure the real-time plant growth by measuring CO_2_ consumption that is robust toward environmental fluctuations. The method implemented through measuring a parameter called *R* and is defined as based on the measured CO_2_ values under two different conditions. The measurement involves calculating/correcting the measured CO_2_ value at a time point with the measurements from the nearby time points making the method to be adaptive and insensitive to external environmental fluctuations. The advantage of the method is that it is made of a simple system and can evaluate plant growth in real time, even in closed systems without precise control of temperature and humidity.

In order to verify the validity of the proposed method, we investigated the relation between the plant growth and variation in light intensity under two different types of illumination, namely, continuous and pulse lights with a closed experimental system.

## Main text

### Experimental system

In this study, arugula (rocket salad, *Eruca sativa*) was used as model plant because of its ease of cultivation (details on growing and experiment conditions given in Additional file 1: A).

**Fig. 1:**
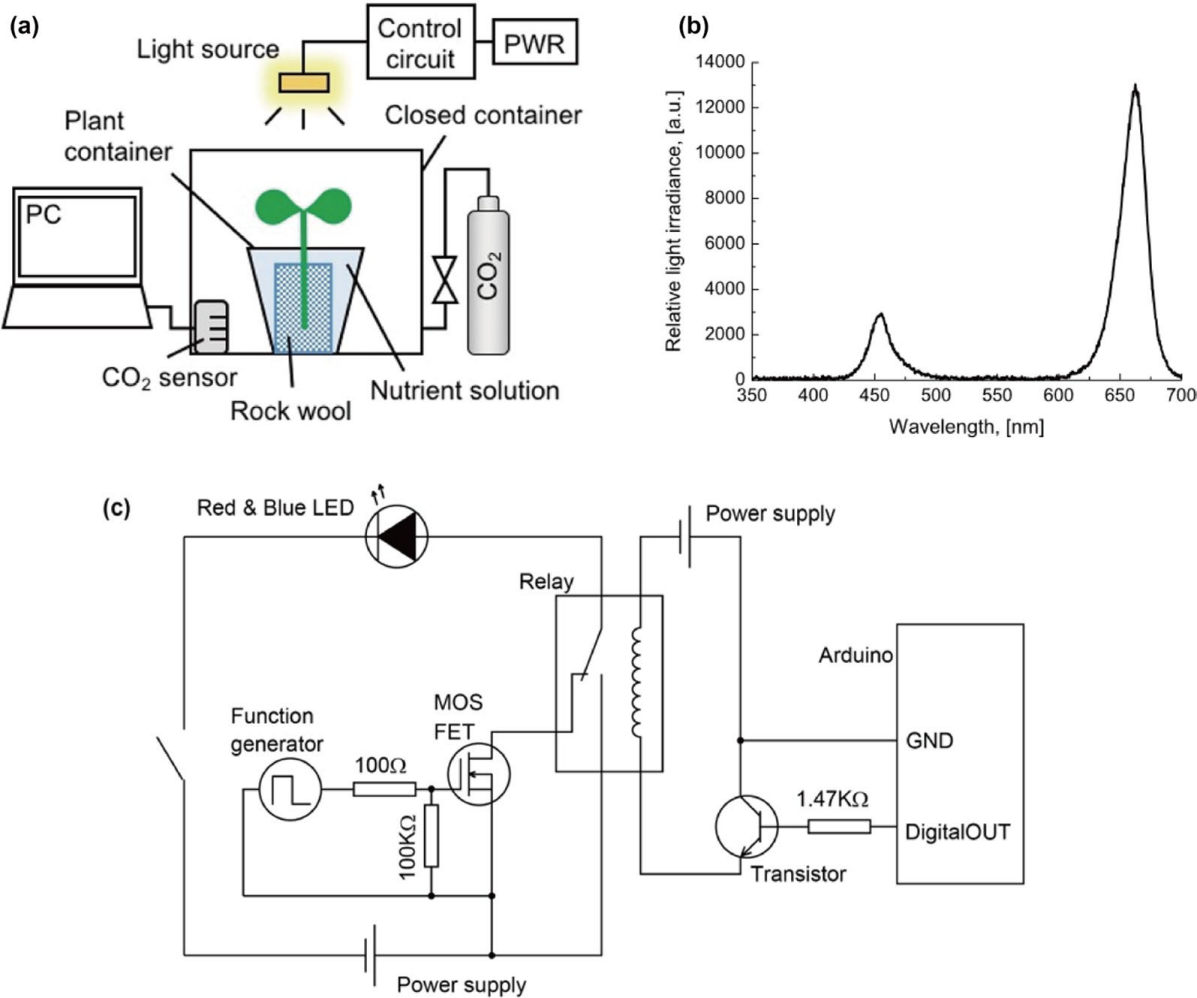
Experimental instrument: **a** experimental system, **b** irradiance of light source as a function of wavelength and c circuit for controlling the PPFD and the pulsed light

Firstly, we made an experimental instrument to measure the CO_2_ consumption during photosynthesis of the plant. Figure 1a shows a schematic of the experimental instrument and the control system with a closed system. By using a closed container, CO_2_ was supplied from an attached CO_2_ cylinder. A Non-Dispersive Infra-Red (NDIR) CO_2_ sensor (TR76Ui; T&D Corporation, Matsumoto, Japan) was placed inside the closed container and connected to an external monitor to measure the variation in CO_2_ concentration each minute. The sensor required a stabilization period of an hour for the CO_2_ concentration to become stable. Therefore, the first cycle of data was discarded. The sensor is also equipped with a thermal sensitive resistor (THA-3001; T&D Corporation, Matsumoto, Japan) and can also be the thermometer in the container. Twelve plant containers of arugula leaves were placed in the closed container, and total CO_2_ consumption of twelve plants was measured.

A light source was positioned above the closed container at a distance of 400 mm from the plants to avoid the increase in temperature. It was comprised of 144 red and 102 blue Light Emitting Diode (LED) bulbs (LH W5AM 1T3T-1 and LH W5AM 3T3U-35, respectively; OSRAM Opto Semiconductors, Regensburg, Germany). The spectral distribution of the light source was measured by a photonic multichannel analyser (Quest X, Konica Minolta, Inc., Tokyo, Japan). Measured relative spectral irradiance of this LED light is shown in Fig. 1b. By integrating measured irradiance and calibrating it with a standard light source, Photosynthetic Photon Flux Density (PPFD) can be evaluated. With the circuit shown in Fig. 1c, the PPFD and the pulsed light can be controlled.

### Principle of the new evaluation method

**Fig. 2:**
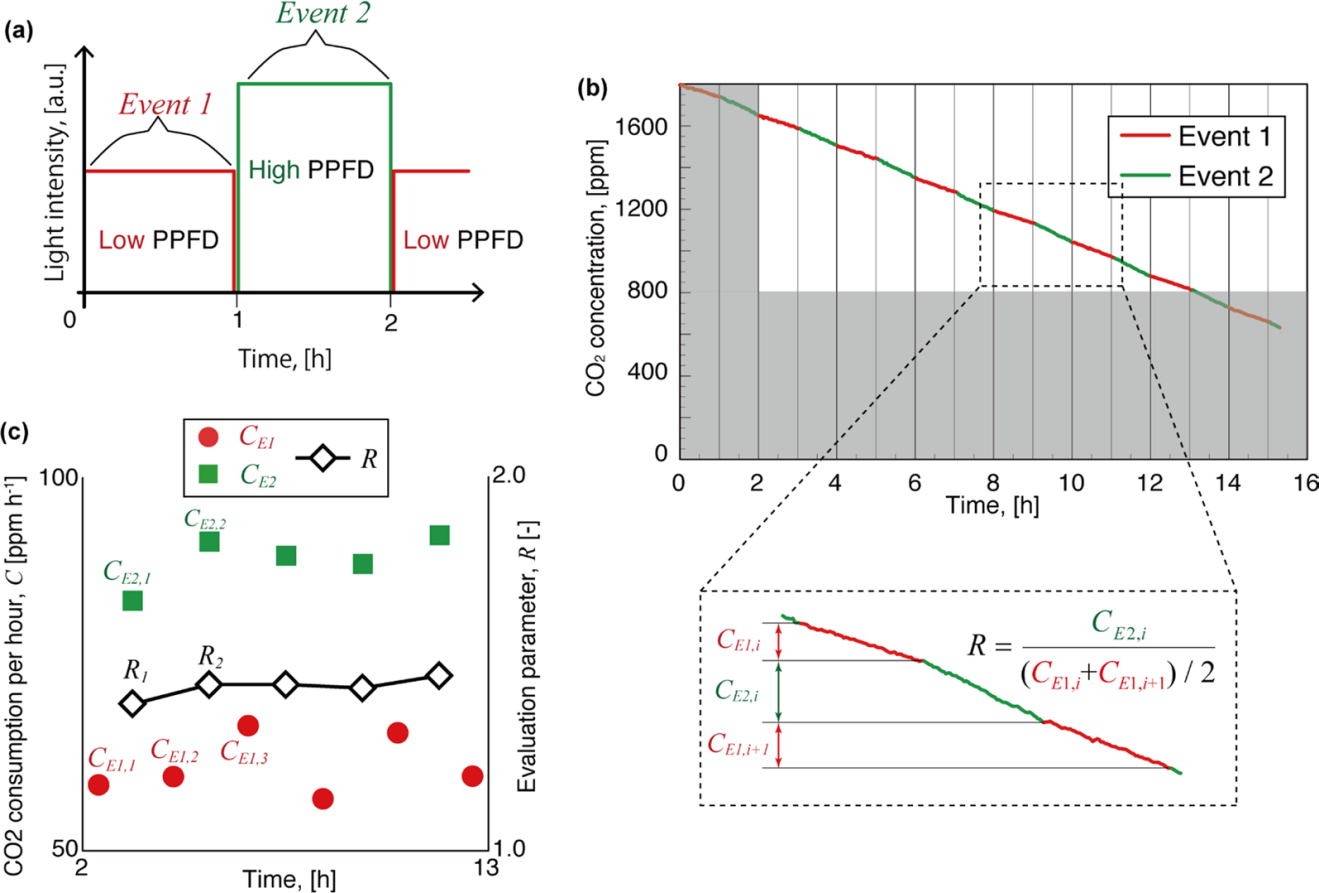
Examples of measured data in a day as a function of hours for **a** rotated condition, **b** CO_2_ consumption and **c** algorithm to calculate the evaluation parameter *R*. The data of CO_2_ consumption from 2 h after the start of the experiment to 800 ppm were used for the evaluation to avoid due to the unstable nature of the sensor (the shaded area in **b** was excluded from the evaluation)

Firstly, Fig. 2a shows the protocol of measurement for the example under alternating light condition of continuous and pulsed illumination. CO_2_ consumption was measured in a day by switching light condition between continuous and pulsed light every hour. The photosynthetic rate changes depending on the carbon dioxide concentration [15]. Although the photosynthetic rate dramatically increases as the CO_2_ concentration increases, the increase of photosynthetic rate saturates under high CO_2_ concentrations.

Figure 2b shows the variation of CO_2_ over a period of 16 h under alternating illumination protocol. In order to minimize the errors that result from the CO_2_ concentration dependence, CO_2_ measured within an extended time under alternate conditions was used as indicated in the Fig. 2b. In addition, the measured temperature and humidity during the experiment given in the supplementary information Additional file 1: Fig. S4 implies that the measured data contain environmental noises which are due to the changes in the temperature and humidity. Therefore, under continuous operation over a long term, there are larger variations in the CO_2_ consumption and thus growth. Instead of using sophisticated equipment to control the environment, we propose an algorithm that reduces the environment related noise from the data.

The parameter *R* used in the new algorithm proposed in this study is a ratio, which is defined as follows:

1 $$ R_{i} = \frac{{C_{E2,i} }}{{(C_{E1,i} + C_{E1,i + 1} )/2}} $$

where, *C* [ppm] is CO_2_ consumption in 1 h. Here the subscripts correspond to the sequential trials obtained under two different conditions or rotations E1 and E2. ‘*E*1*’* and *‘E*2’ mean 'Event 1' and 'Event 2’, respectively. The indices in subscripts, *i* or *i* + 1 correspond to the counts of rotation of the Events 1 and 2 that occur alternatively. As can be seen from Fig. 2b, the concentration of CO_2_ within the closed chamber is not constant but gradually decreasing which in turn may affect the CO_2_ consumption itself. In order to reduce the effect of the noise, average of the data of Events *E*1*, i* and *E*1*, i* + 1 occurring at the denominator of Eq. 1 was used as a factor to compare with the data of an Event *E*2*, i*. Having a ratio of CO_2_ measured under two different illumination conditions (Events, *E*1 and *E*2) makes the ratio almost insensitive to the variations of CO_2_ over the long time. Therefore, the proposed method can compensate (or adapt to) for the local environmental fluctuations within a relatively short time of a few cycles of data acquisition. As long as there are no drastic variations in the environment, this method can robustly evaluate or correct for the effect of environmental conditions on the plant growth. Figure 2c shows the calculated example of *R* for the example data shown in Fig. 2b. As shown in Fig. 2c this proposed method can measure over sufficient number of rotations and thus having a fairly sufficient number of *R* values within a day.

### Results and discussions

**Fig. 3:**
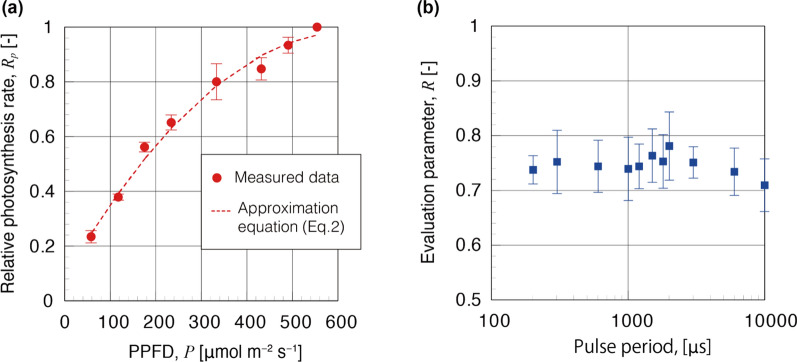
Measurement result using the proposed method: **a** the relationship between the PPFD and relative photosynthesis rate and **b** effect of pulsed light on the plant growth. In **a** red dashed line shows the approximation equation for our data. The error bars in **a** and **b** are the standard deviation of the measured data

Firstly, we measured the relationship between the PPFD and relative photosynthesis rate by using our adaptive algorithm with the closed system. This experiment was conducted by fixing Event 1 being the illumination under light PPFD of 553 µmol m^−2^ s^−1^, and setting the Event 2 as being under a variable PPFD. Here the relative photosynthesis rate was normalized with the PPFD of Event 1 as 1.0. The measurement was performed over one day for each of the PPFD conditions, and the error bars shown in Fig. 3a are the standard deviations for the measured data. We have found a good agreement with the reference data (Jie He et al. [10]) (details on the comparison and validity of this method in Additional file 1: B).

Finally, we investigated the effect of pulse periods of light source on plant growth using our method. Figure 3b shows the evaluation parameter *R* against the pulse period of the light source. In this experiment, constant light was fixed and periods of the pulsed light was varied. The duty ratio for the pulsed light was set to 50%. The measurement was performed for 4 days for each of the pulse period conditions. The error bars in Fig. 3b correspond to standard deviation.

In addition, PPFD difference between constant and pulsed illuminations should be properly considered in order to evaluate the efficiency of the method. Following simple approximation equation as shown in Fig. 3a, the dashed line, corresponded to the fitted line as determined by the least square method.

2 $$ R_{p} (P) = - 2.17 \times 10^{ - 6} \times P^{2} + 2.80 \times 10^{ - 3} \times P + 9.65 \times 10^{ - 2} , $$

where the *R*_*p*_ [–] is the relative photosynthesis rate and the *P* [µmol m^−2^ s^−1^] is the PPFD.

The value of PPFD decreased from 113 to 75.3 µmol m^−2^ s^−1^ when switching from continuous light to pulsed light. The decrease in PPFD led to a decreased photosynthetic rate calculated to be about 0.77 [= *R*_*p*_ (75.3)/*R*_*p*_ (112)]. The averaged value of *R* over all experimental range of pulse periods was about 0.75. Therefore, strictly speaking, the evaluation parameter *R*, ratio characterizing the effect of pulsed light and continuous light, would reflect a change in photosynthesis efficiency due to the change in PPFD.

Tennessen et al. [20] using tomato leaves found that when photons were provided during 1.5 μs of pulsed light followed by 148.5 μs dark periods, the photosynthesis was the same as in the continuous provided the integrated photons during the pulsed light are equivalent. This report suggested that the photons in pulses of 100 μs or shorter are absorbed and stored in the reaction centers to be used in electron transport during the dark period. Although the types of plants and used pulse periods range are different from [20], our measurement results agree with their results. In addition, Jao and Fang [21] have investigated the effects of pulsed light on the growth of potato plantlets and energy savings by using LEDs compared to the use of conventional tubular fluorescent lamps. They showed that the pulsed LEDs at 720 Hz and 50% duty ratio with 16-h light/8-h dark photoperiod could produce the highest photosynthesis growth rate, and LEDs at 180 Hz and 50% duty ratio with 16-h light/8-h dark photoperiod would be the best choice when considering the efficiency of the yield with respect to energy consumption. Optimal light source conditions considering of photosynthesis rate and energy consumption differ depending on the types of plant [20–23]. It would be an industrial advantage if the optimal light source conditions could be investigated and realized by using pulsed light with LEDs.

## Limitations

Our results suggest that our evaluation method can evaluate the effect of rotated condition on plant growth by removing the environmental noises without precise control of temperature and humidity. However, it is necessary to investigate what kind of light source can make high photosynthesis rate and saving energy consumption for various types of plants. In addition, considering the experimental principle of this study, it is not possible to evaluate the results in the case where R changes rapidly within one cycle.

## Supplementary Information

**Additional file 1:** **Figure S1.** Plant sample: (a) Arugula before the experiments and (b) twelve plant containers of arugula placed in closed container. **Table S1.** Ingredients in the nutrient solution. **Figure S2.** Comparison of the measured CO2 consumption against the weight change of argula with red squares indicating the measurement data and the red line indicating the least squares fit with significant correlation (correlation coefficient r =0.995). **Figure S3.** Effect of rotation time on measurement of the evaluation parameter R. **Figure S4.** Examples of measured data of temperature and humidity in a day during experiment. Figure S5 Comparison of our measurement results and the reference data [10].

## Data Availability

The datasets used and/or analyzed during the current study are available from the corresponding author on reasonable request.

## Abbreviations

LED: Light emitting diode
PPFD: Photosynthetic photon flux density
