# Supplementary material for: A simple adaptive difference algorithm with CO2 measurements for evaluating plant growth under environmental fluctuations
Source: BMC Res Notes. 2022 Feb 14;15:48. doi: 10.1186/s13104-022-05929-0 (PMC8842854; doi:10.1186/s13104-022-05929-0)
Supplement: Supplementary file 1 — Additional file 1: Figure S1. Plant sample: (a) Arugula before the experiments and (b) twelve plant containers of arugula placed in closed container. Table S1. Ingredients in the nutrient solution. Figure S2. Comparison of the measured CO2 consumption against the weight change of argula with red squares indicating the measurement data and the red line indicating the least squares fit with significant correlation (correlation coefficient r =0.995). Figure S3. Effect of rotation time on measurement of the evaluation parameter R. Figure S4. Examples of measured data of temperature and humidity in a day during experiment. Figure S5 Comparison of our measurement results and the reference data [10]. [file 13104_2022_5929_MOESM1_ESM.pdf]

Additional materials

A simple adaptive difference algorithm with CO_2 measurements for evaluating plant growth under environmental fluctuations

Hiroki Gonome[1], Jun Yamada[2], Norito Nishimura[2], Yuta Arai[2], Minoru Hirai[2], Naoki Kumagai[2], Uma Maheswari Rajagopalan[2], Takahiro Kono[2*]

[1] Department of Mechanical System Engineering, Yamagata University, Yamagata, 992-8510, Japan

[2] Department of Mechanical Engineering, Shibaura Institute of Technology, 3-7-5 Toyosu, Koto-ku, Tokyo 135-8548, Japan

*Corresponding author. E-mail address: kono.takahiro.f3@sic.shibaura-it.ac.jp

A: Plant condition

In this study, arugula (rocket salad, *Eruca sativa*) was used as model plant. Prior to the start of the experiment, seeds were sown on rock wool, watered for 10 days in a cup, following which they sprouted. After sprouting, the plants were transferred individually into the cups to hold each plant and placed inside a closed container shown below. Nutrient solution was added to the plant cups. Figure 1 shows photographs of the plants, arugula before the experiments and twelve plant containers of arugula within the closed container.

(a) (b)

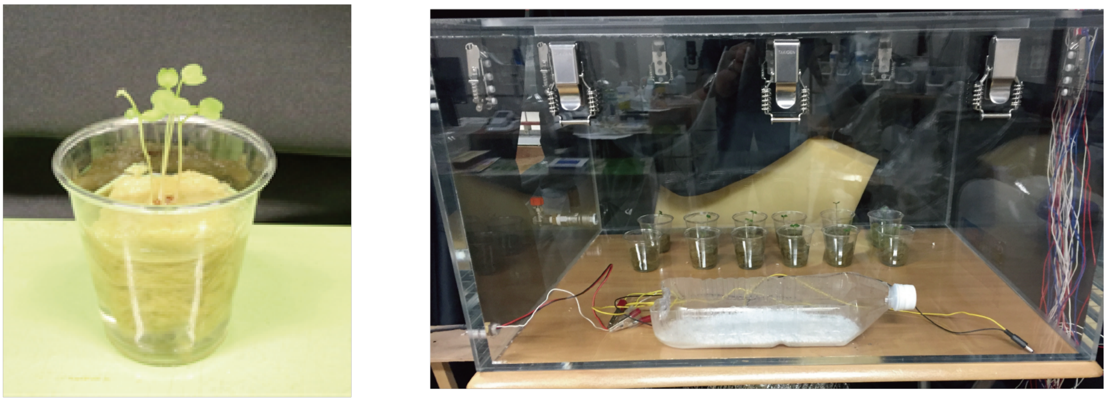

Figure S1 Plant sample: (a) Arugula before the experiments and (b) twelve plant containers of arugula placed in closed container.

Fertilizers (OAT House 1 and 2; OAT Agrio Co., Ltd., Tokyo, Japan) were used to make the nutrient solution for nutriculture. Amounts of 1.5 g and 1.0 g of OAT House 1 and OAT This nutrient solution was diluted with water until the electrical conductivity was 0.1 S m^{-1}, according to the fertilizer manufacturer’s instructions. The solution’s electrical conductivity was determined using a water quality measurement instrument (Model-7200; FUSO Chemical Co., Ltd., Tokyo, Japan). Table 1 lists all the ingredients used in the nutrient solution, as derived from the OAT House 1 and 2 / water mix.

Table 1 Ingredients in the nutrient solution

Ingredient	Amount [ppm]
Total nitrogen	100.000
(Ammoniacal nitrogen)	8.846
(Nitrate-nitrogen)	89.615
(Urea($CO(NH_2)_2$))	1.539
Phosphorus pentoxide	46.154
Potassium oxide (K_2O)	155.769
Lime (CaO)	88.462
Magnesium oxide (MgO)	23.077
Manganese(II) oxide	0.577
Boron trioxide (B_2O_3)	0.577
Iron (Fe)	1.038
Copper (Cu)	0.012
Zinc (Zn)	0.012
Molybdenum (Mo)	0.012

B: Examination of validity of experimental method

B-1: Relationship between CO_2 consumption and plant growth

To examine whether plant growth can be evaluated from CO_2 consumption, correlation between incremental growth and CO_2 consumption was measured for arugula. Such experiments have been conducted for a long time, but this experiment was also performed to show the sealing performance of the instrument made here. Arugula plants were cultivated in the closed container, and CO_2 depletion in the container was measured. The mass of the plant was measured using an electronic balance (EK-410i; A&D Co. Ltd., Tokyo, Japan). The carbon masses of arugula were evaluated from both the measured plant mass and the measured CO_2 consumption.

Figure 2 shows the calculated mass of carbon in arugula. The mass of carbon in arugula calculated from CO_2 consumption was highly correlated to the mass of carbon in arugula calculated from the mass of arugula. The correlation coefficient r = 0.995. This result showed that it was appropriate to evaluate the growth state using CO_2 consumption in this system.

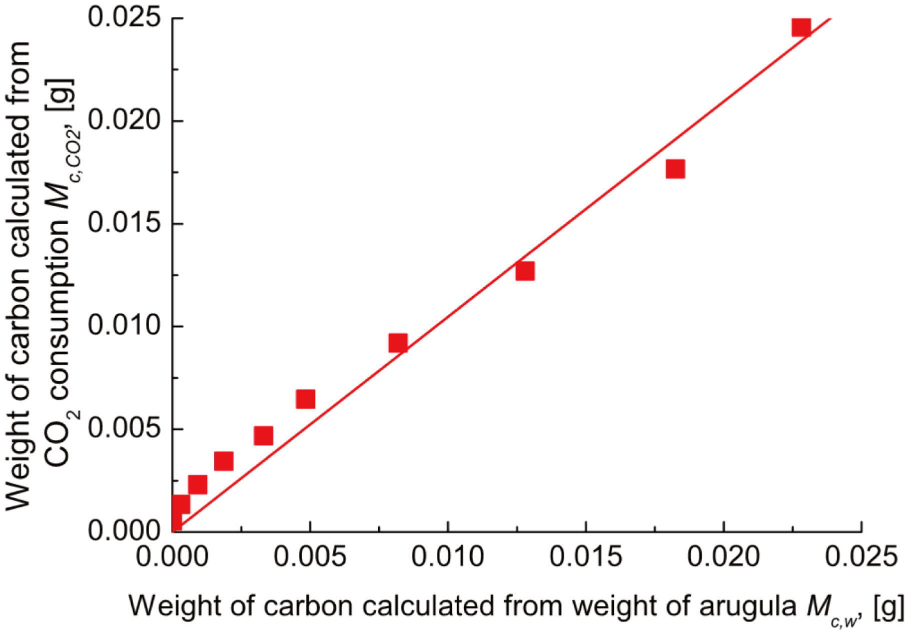

Figure S2 Comparison of the measured CO_2 consumption against the weight change of argula with red squares indicating the measurement data and the red line indicating the least squares fit with significant correlation (correlation coefficient *r* = 0.995)

B-2: Effect of rotation time on evaluation

In order to verify the appropriateness of the rotation time, we used rotation times of continuous (Event 1) and pulsed light (Event 2) varying from every 10 to 120 min and verified a suitable range of rotation time. As part of the standard condition, we cultivated the plants using constant light. We used one hour as the rotation time in the experiments.

Figure 3 shows the effects of rotation time on the ratio of CO_2 consumption *R*. In this method, it is necessary to make measurement only on the influence of change of the Events independent of the rotation time. However, for a range of rotation time from 10 min to 30 min, *R* was unstable, making the rotation time to be too short. On the other hand, when the rotation time was over 40 min, *R* was almost constant, and the measurement was stable. This means that

the shortest rotation time to evaluate the growth condition was found to be 40 min. In this experiment, although we did not verify a longer limit of rotation time, a rotation time over 120 min is not recommended because prolonged environment changes could affect the evaluation parameter *R* very much. Therefore, the suitable range of rotation time is from 40 min to 120 min.

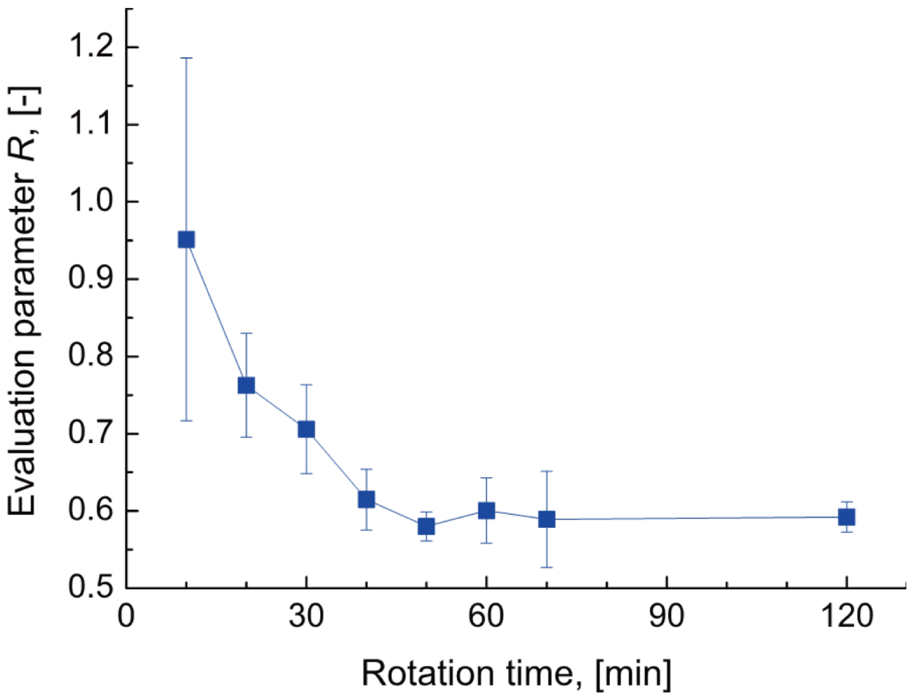

Figure S3 Effect of rotation time on measurement of the evaluation parameter *R*.

B-3: Comparison of our measured results and reference data

To validate our evaluation method, we measured the relationship between the PPFD and relative photosynthesis rate by using our developed system. In our measurement, the cultivation temperature was about 30.0 ± 1.0 °C and humidity was about 80 ± 5% in all of the experiments. An example of the measured temperature and humidity in a day during this experiment was shown in Figure 4. On the other hand, the reference experiments using plants after twelve days of germination (Jie He et al. [10]) that controlled the temperature precisely, the temperatures

were in the range of 29.6 ± 0.1 ˚C, and they measured the photosynthesis rate with an open infrared gas analysis system with a 6 cm^2 chamber (LI-6400, Biosciences, US).

Our experiment was conducted by fixing Event 1 of continuous illumination with a light level of 553 μmol m^{-2} s^{-1}, and setting the Event 2 as a variable PPFD. Therefore, for the latter, a relative photosynthesis rate normalized to the PPFD of Event 1 was defined summarize the results of them in Figure 5.

As shown in Figure 5, our measurement result was good agreement with the reference data. Even if environmental noise was included as shown in Figure 4, the effect of environmental noise on the evaluation of photosynthetic rate could be reduced by using our proposed method.

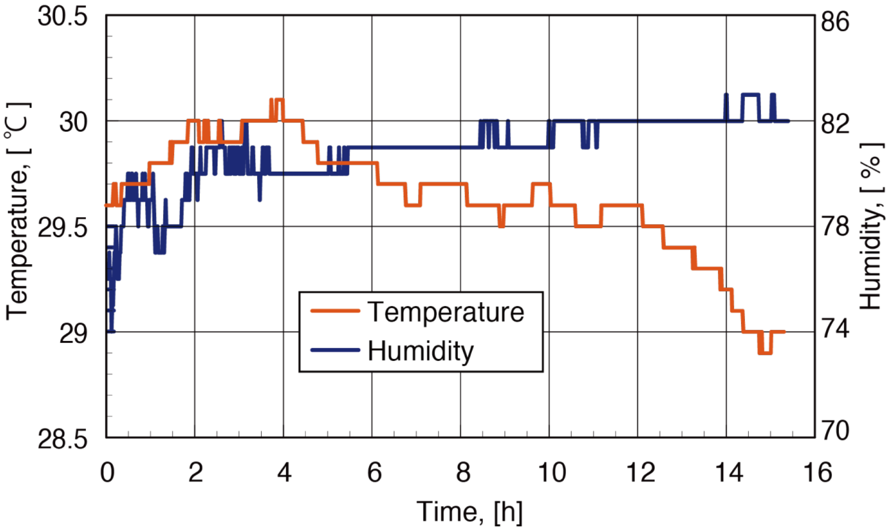

Figure S4 Examples of measured data of temperature and humidity in a day during experiment.

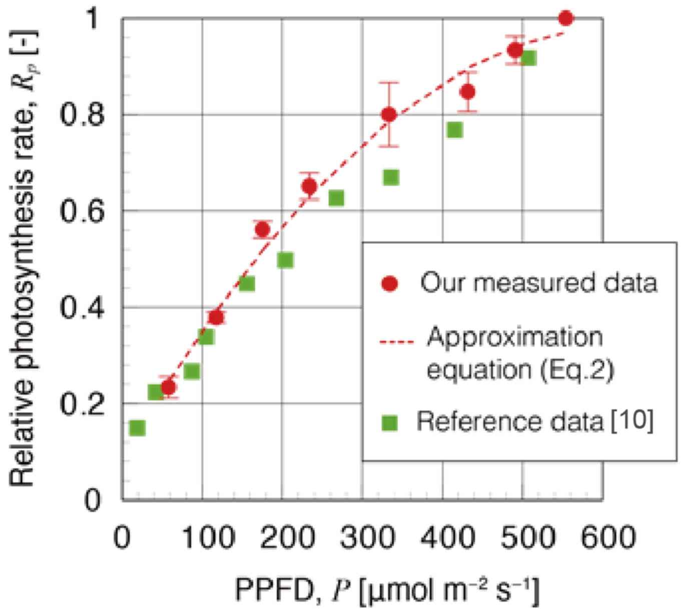

Figure S5 Comparison of our measurement results and the reference data [10].
